# Phase Evolution and Synthesis of Be_12_ Nb Intermetallic Compound in the 800–1300 °C Temperature Range

**DOI:** 10.3390/ma18122915

**Published:** 2025-06-19

**Authors:** Sergey Udartsev, Inesh E. Kenzhina, Timur Kulsartov, Kuanysh Samarkhanov, Zhanna Zaurbekova, Yuriy Ponkratov, Alexandr Yelishenkov, Meiram Begentayev, Saulet Askerbekov, Aktolkyn Tolenova, Manarbek Kylyshkanov, Mikhail Podoinikov, Ainur Kaynazarova, Oleg Obgolts

**Affiliations:** 1 Department of General Physics, Satbayev University, Almaty 050032, Kazakhstan; udartsevsv@ulba.kz (S.U.); kenzhina@physics.kz (I.E.K.); tima@physics.kz (T.K.); zzha@physics.kz (Z.Z.); ponkratov@nnc.kz (Y.P.); aleksandrelisenkov282@gmail.com (A.Y.); m.begentayev@satbayev.university (M.B.); askerbekov@physics.kz (S.A.); aktolkyn.tolenova@physics.kz (A.T.); 2 Ulba Metallurgical Plant JSC, Ust-Kamenogorsk 070005, Kazakhstan; kylyshkanovmk@ulba.kz (M.K.); podoinikovma@ulba.kz (M.P.); k.ainura0572@gmail.com (A.K.); obgoltsoj@ulba.kz (O.O.); 3 Materials Testing Department, Institute of Atomic Energy Branch of National Nuclear Center of the Republic of Kazakhstan, Kurchatov 071100, Kazakhstan

**Keywords:** Be_12_Nb, intermetallics, synthesis, powder metallurgy, beryllides

## Abstract

Beryllium-based intermetallic compounds, such as Be_12_Nb, are attracting growing interest for their high thermal stability and potential to replace pure beryllium as neutron reflectors and multipliers in both fission and future fusion reactors, with additional applications in metallurgy, aerospace, and hydrogen technology. The paper presents the results of an investigation of the thermal treatment and phase formation of the intermetallic compound Be_12_Nb from a mixture of niobium and beryllium powders in the temperature range of 800–1300 °C. The phase evolution was assessed as a function of sintering temperature and time. A nearly single-phase Be_12_Nb composition was achieved at 1100 °C, while decomposition into lower-order beryllides such as Be_17_Nb_2_ occurred at temperatures ≥1200 °C, indicating thermal instability of Be_12_Nb under vacuum. Careful handling of sintering in low vacuum minimized oxidation, though signs of possible BeO formation were noted. The findings complement and extend earlier reports on Be_12_Nb synthesis via plasma sintering, mechanical alloying, and other powder metallurgy routes, providing broader insight into phase formation and synthesis. These results provide a foundation for optimizing the manufacturing parameters required to produce homogeneous Be_12_Nb-based components and billets at an industrial scale. Additionally, they help define the operational temperature limits necessary to preserve the material’s phase integrity during application.

## 1. Introduction

Beryllium-based intermetallic compounds [1,2], particularly those of the Be_12_M type (where M = Ti, Cr, V, Zr, etc.), have attracted increasing interest due to their high thermal stability, low swelling, and favorable neutron breeding properties, making them promising candidates for use as neutron multipliers in future fusion facilities such as ITER and DEMO [3,4,5,6,7]. DEMO-type reactors are projected to operate under more extreme conditions than ITER, including higher neutron flux [8,9,10] and sustained high temperatures, requiring structural and functional materials with enhanced oxidation resistance [11,12], high melting points, and low tritium retention [13,14].

The intermetallic compound Be_12_Nb has been proposed as a potential alternative to pure beryllium for high-temperature applications in both nuclear [15] and aerospace systems [16,17] due to its theoretically high thermal stability [18], corrosion resistance, etc. Additional interest has emerged around its potential use in hydrogen-related technologies, such as solid-state hydrogen storage [19]. However, it must be noted that Be_12_Nb has not yet seen practical implementation due to challenges in synthesis, phase stability, and limited mechanical property data.

**Figure 1:**
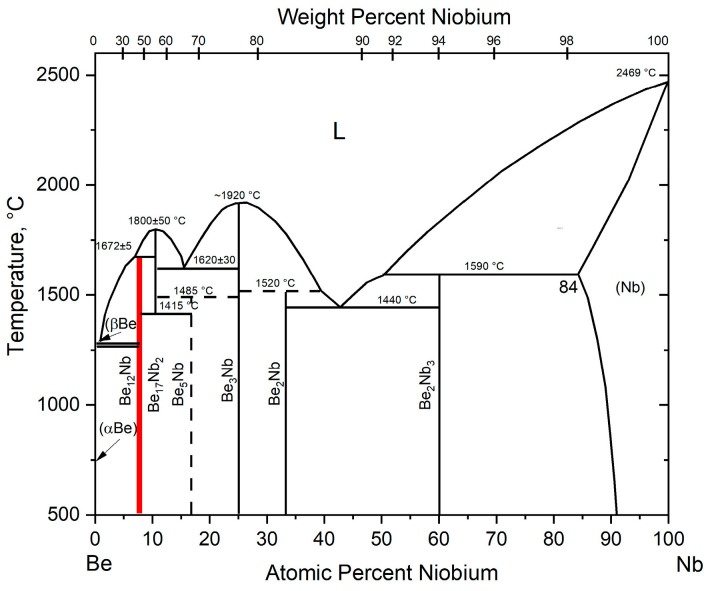
Be-Nb phase diagram.

The phase diagram of the beryllium–niobium (Be–Nb) system at atmospheric pressure is given in [20] and shown in Figure 1.

Six intermetallic compounds are formed in the Be–Nb system: Be_12_Nb, Be_17_Nb_2_, Be_5_Nb, Be_3_Nb, Be_2_Nb, and Be_2_Nb_3_.

The compound Be_12_Nb is formed via a peritectic reaction at 1672 °C from the liquid phase and the compound Be_17_Nb_2_. According to the diagram, the intermetallic Be_17_Nb_2_ melts congruently at 1800 °C. At 1415 °C, it decomposes to form Be_12_Nb and Be_5_Nb. The compound Be_5_Nb is presumably formed via a peritectoid reaction at 1485 °C from two intermetallics: Be_17_Nb_2_ and Be_3_Nb. The intermetallic compound Be_3_Nb has the highest melting point in the system, equal to 1980 °C. It melts congruently and does not undergo further transformations down to room temperature. The beryllide Be_2_Nb is also formed via a peritectic reaction from the liquid phase and the intermetallic Be_3_Nb. Finally, the intermetallic Be_2_Nb_3_ is formed via a peritectic reaction from the solid solution of niobium in beryllium and the liquid phase at 1590 °C. There are also two eutectic reactions in the system: Be_17_Nb_2_ + Be_3_Nb and Be_2_Nb + Be_2_Nb_3_, with eutectic temperatures of 1620 and 1440 °C, respectively.

The synthesis of Be_12_Nb is complicated by several intrinsic material factors: its high melting point (1672 °C) [20], the large disparity between the melting points of its constituent elements (Be: 1283 °C; Nb: 2468 °C), the volatility and chemical reactivity of beryllium, and the tendency for melt contamination or crucible interaction under high-temperature conditions. These difficulties make conventional melting methods impractical, thereby driving interest in solid-state sintering routes, such as powder metallurgy, to synthesize Be_12_Nb.

Obtaining large-sized billets or workpieces from beryllium intermetallics using conventional alloying methods faces several clear challenges. The main drawback is the inability to achieve a single-phase Be_12_Nb structure. Due to the relatively high melting point of Be_12_Nb (1672 °C) and the significant difference in the melting points of beryllium (1285 °C) and niobium (2468 °C), a key challenge is identifying a reliable melting method that also suppresses the evaporation of beryllium, which has a high vapor pressure during the melting process. The high reactivity of beryllium necessitates melting in crucibles made of beryllium oxide; otherwise, the melt becomes contaminated with impurities from the crucible material. Additionally, the high melting temperatures of the final product require overheating the melt to prevent early crystallization when it comes into contact with the mold or necessitate preheating the mold. The low casting characteristics of beryllium intermetallics lead to significant shrinkage cavities, porosity, and cracks. For this reason, the manufacturing of large-sized billets and components from beryllides is usually produced using vacuum pressure solid-state sintering methods.

Several approaches have been explored to synthesize Be_12_Nb. In [21] used plasma sintering at 900–1000 °C was used, and observed that increased Be_12_Nb phase formation occurred with extended sintering durations. In a follow-up study, [22] compared sinterability and thermal stability of Nb- and V-based beryllides, confirming that prolonged sintering times enhanced the Be_12_Nb fraction but did not achieve phase-pure material [19]. Similarly, [23] demonstrated Be_12_Nb formation via mechanical alloying and annealing, although notable contamination from tungsten carbide milling media was observed. Sputter deposition methods have also been used to investigate phase stability and composition ranges of Be_12_Nb thin films [24,25,26,27], revealing a stoichiometric window and potential for metastable phase formation. Hot isostatic pressing (HIP) has shown success in synthesizing dense Be_12_Nb samples, though later studies have indicated potential for embrittlement and grain boundary degradation [28,29].

In previous work, the authors of the present study investigated the synthesis of Be_3_Nb, identifying a sequence of intermediate beryllide phases and optimal conditions for single-phase formation at 1300 °C [30]. Building on that, the current study focuses on the synthesis of Be_12_Nb applied to a stoichiometric mixture of pre-compacted beryllium and niobium powders within the temperature range of 800 °C and 1300 °C. The aim is to determine the optimal conditions for forming a single-phase Be_12_Nb compound as a function of sintering temperature and time. The research findings can be applied in the development of vacuum hot pressing or plasma sintering processes for producing single-phase Be_12_Nb billets.

## 2. Materials and Methods

High-purity PTB-56 grade beryllium powder and NbP-3b grade niobium powder, both produced by UMP JSC (Ust-Kamenogorsk, Kazakhstan), were used in this study. These industrially manufactured powders are known for their high chemical purity and were selected based on characteristics similar to those reported in [30].

For the research, PTB-56 grade beryllium powder and NbP-36 grade niobium powder [31] were used, both produced by JSC “Ulba Metallurgical Plant,” Ust-Kamenogorsk, Kazakhstan. The chemical compositions of the beryllium and niobium powders are provided in Table 1 and Table 2.

**Figure 2:**
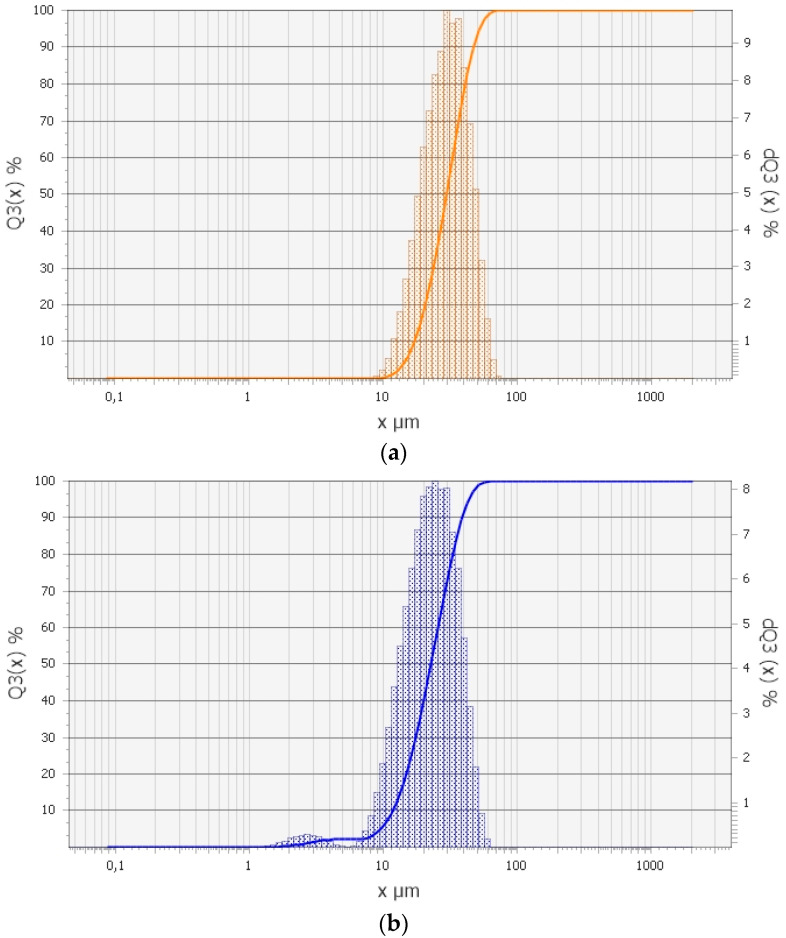
Fractional composition of powders: (**a**) NbP-3b niobium; (**b**) PTB-56 beryllium.

Particle size analysis was performed using an Analysette 22 NanoTec (Fritsch GmbH, Markt Einersheim, Germany) laser particle sizer. The particle size distribution curve of Nb and Be powders is given in Figure 2.

Figure 2a shows that the particle sizes of niobium powder range from 10 to 70 µm, with an average effective particle size of 31.27 µm. Figure 2b demonstrates that the particle size distribution of beryllium powder exhibits two distinct regions. Approximately 4.0% by mass of the beryllium powder particles have sizes ranging from 1.5 to 6 µm, with an average size of about 1.8 µm in this range, referred to as the fine fraction. The remaining particles range in size from 6 to 60 µm. Overall, the average effective particle size of the beryllium powder is 23.67 µm.

**Figure 3:**
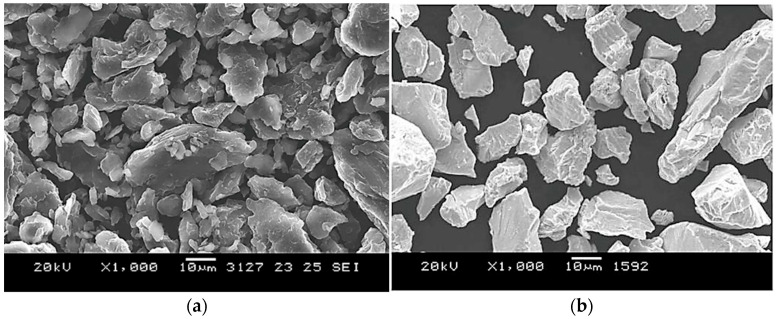
SEM images of powder particles: (**a**) beryllium; (**b**) niobium.

SEM images of the powder particles obtained using an electron microscope at ×1000 magnification are shown in Figure 3.

As seen, the beryllium powder particles predominantly have a round, flaky shape without internal voids, while the niobium powder particles exhibit an angular shape with sharp edges and flat faces, also without internal voids.

**Figure 4:**
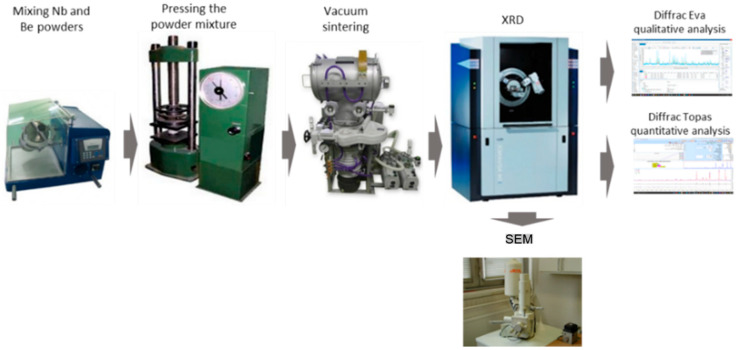
Schematic representation of the experimental workflow for the synthesis of the Be_12_Nb binary intermetallic compound.

To synthesize Be_12_Nb, beryllium and niobium powders were mixed in a stoichiometric ratio corresponding to 53.79 wt.% Be and 46.21 wt.% Nb. Mixing was carried out for at least 1 h using a Turbula C 2.0 turbulent mixer, Willy A. Bachofen AG, Basel, Switzerland. The mixed powder was then compacted using a P-10 hydraulic press, Carver, Inc., Wabash, IN, USA with a maximum load of 100 kN, producing cylindrical briquettes with diameters of 14 mm and heights of approximately 20 mm. A schematic representation of the overall experimental procedure is provided in Figure 4.

Sintering was conducted in a vacuum furnace in the temperature range of 800 °C to 1300 °C, in 100 °C increments. For each temperature, holding durations of 1, 2, and 3 h were used. The heating rate was maintained at 15–20 °C/min, and vacuum pressure during sintering did not exceed 2 × 10^−3^ Torr. No external pressure was applied during sintering, and the samples were left to cool naturally in the furnace after the holding period. A total of 22 samples were synthesized under varying time-temperature conditions.

After sintering, each sample was manually ground to a powder with an average particle size of 10–20 μm for structural and phase analysis. X-ray diffraction (XRD) analysis was performed using a Bruker D8 Advance Eco diffractometer, Bruker, Billerica, MA, USA. Scanning parameters were consistent with those used in [30]. The quantitative phase analysis was performed using the Rietveld method [32] with the TOPAS 6.0 software [33,34] and crystal structure data from the *.cif files of niobium beryllides.

**Figure 5:**
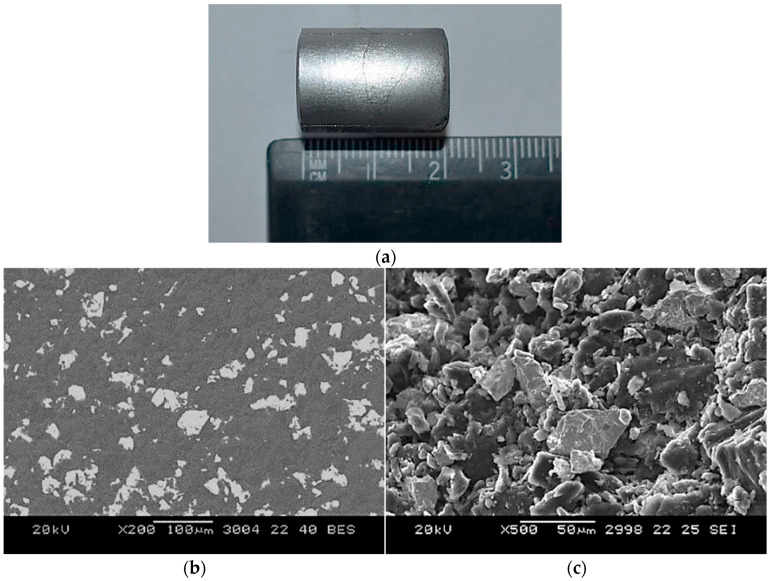
Appearance and microstructure of the Be–Nb powder compact prior to sintering: (**a**) Densely pressed cylindrical briquette; (**b**) SEM image in backscattered electron (BSE) mode; (**c**) SEM image in secondary electron (SE) mode.

Microstructural characterization was conducted using a JEOL JSM-5610 scanning electron microscope, JEOL, Tokyo, Japan. Both the backscattered electron (BSE) mode, for compositional contrast, and the secondary electron (SE) mode, for surface topography, were used. Representative results showing the pressed briquette and SEM images at different magnifications are presented in Figure 5. The images confirm that beryllium (darker regions) and niobium (lighter regions) powders were in tight mechanical contact before sintering.

## 3. Results

**Figure 6:**
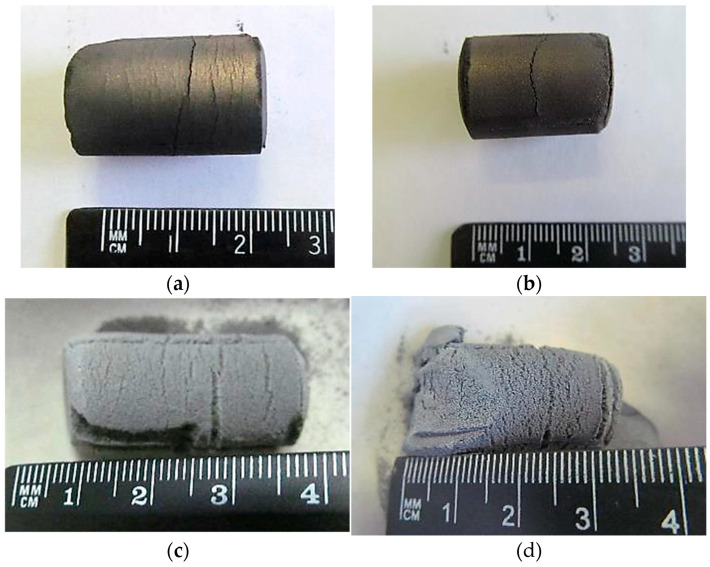
Appearance of briquettes after sintering: (**a**) 2 h at 800 °C; (**b**) 3 h at 800 °C; (**c**) 2 h at 1300 °C; (**d**) 6 h at 1300 °C.

Sintering of pressed briquettes composed of niobium and beryllium powders led to a visible increase in volume and a loosening of structural integrity as the reaction progressed. Figure 6 illustrates briquettes sintered at different temperatures: (a) and (b) after 2 h and 3 h at 800 °C, respectively, and (c) and (d) after 2 h and 6 h at 1300 °C. At higher sintering temperatures, more pronounced expansion was observed. In contrast, samples sintered at 800 °C exhibited no substantial morphological changes.

The volumetric expansion of the briquettes can be attributed to two main factors. First, increased internal pressure due to residual gases trapped in the porous matrix may cause mechanical expansion upon heating. Second, and more significantly, the formation of intermetallic compounds during sintering leads to substantial lattice volume increases. As shown in Table A1, intermetallic phases such as Be_12_Nb (231.3 Å^3^) possess unit cell volumes far exceeding those of the starting elements—niobium (36.15 Å^3^) and beryllium (16.22 Å^3^). This transformation, caused by the rearrangement of the crystal structure from elemental powders to larger-volume beryllide structures, underpins the observed macroscopic swelling. Furthermore, as the powders react and form new phases, the initial compacted structure becomes disrupted, resulting in visible loosening of the briquettes.

The results of X-ray phase analysis and quantification of niobium beryllide phases formed in each temperature–time range are presented in the following subsections.

### 3.1. Synthesis at 800 °C

**Figure 7:**
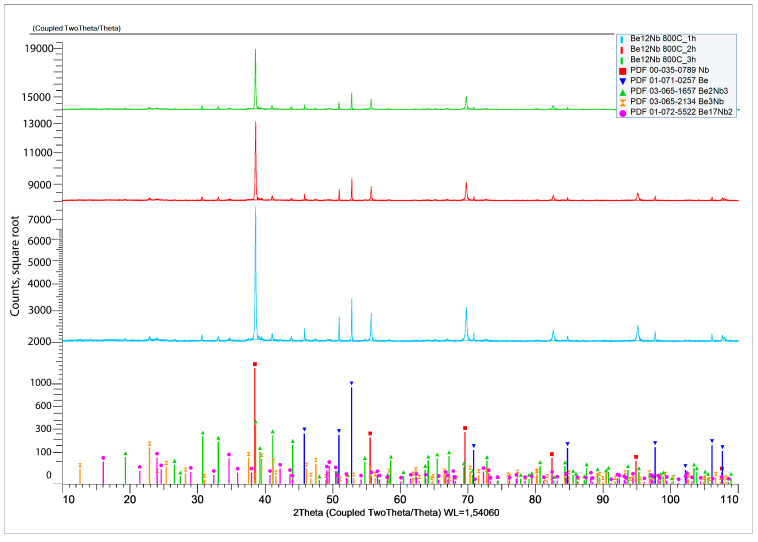
X-ray diffraction patterns after sintering at 800 °C.

XRD results after sintering at 800 °C for 1, 2, and 3 h are shown in Figure 7.

**Figure 8:**
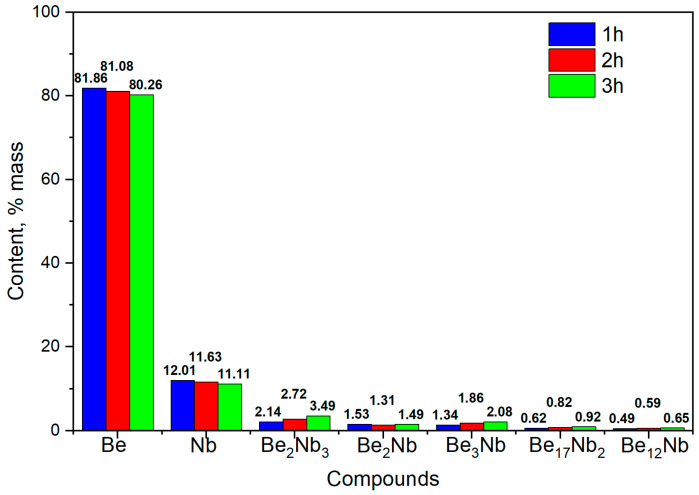
Phase composition of powders after sintering at 800 °C for 1 h (blue), 2 h (red), and 3 h (green).

At the initial stage of synthesis at 800 °C, the diffraction patterns are dominated by peaks corresponding to the unreacted beryllium and niobium. The intensity of peaks associated with intermetallic compounds (Be_12_Nb, Be_17_Nb_2_, Be_3_Nb, Be_2_Nb, Be_2_Nb_3_) remains significantly lower, indicating limited phase formation. The samples predominantly consist of beryllium (~80 wt.%) and niobium (~12 wt.%), as shown in the quantitative analysis using TOPAS 6.0 software (Figure 8).

The low intensity of beryllide peaks suggests that either the activation energy at this temperature is insufficient to drive significant reaction, or the reaction kinetics are too slow to form appreciable amounts of intermetallics within the 1–3 h range. Only slight synthesis of beryllides with Be/Nb atom ratios < 3 or =3 was observed. Nb_3_Be_2_ content remained under 4 wt.%, while Be_2_Nb and Be_3_Nb did not exceed 2 wt.% each.

**Figure A1:**
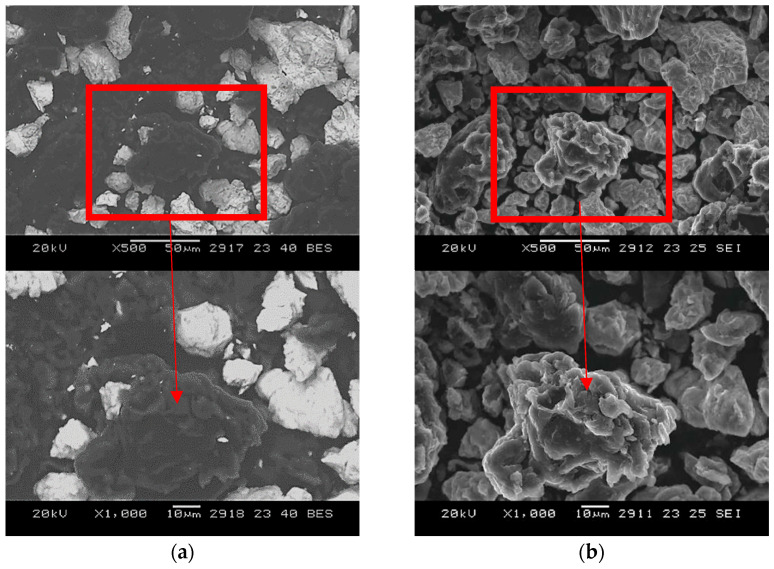
SEM images of niobium beryllide powders synthesized at 800 °C for 2 h: (**a**) BSE mode; (**b**) SE mode.

SEM images of powders of niobium beryllides synthesized at 800 °C for 2 h, taken in SE and BSE modes at various (×500 and ×1000) magnifications, are shown in Figure A1.

SEM images of powders sintered at 800 °C for 2 h confirm the limited reaction progress. Particles of the original powders remain distinct, with little to no evidence of morphological transformation. This further supports the conclusion that at 800 °C, diffusion is insufficient to promote extensive phase development.

### 3.2. Synthesis at 900 °C

**Figure 9:**
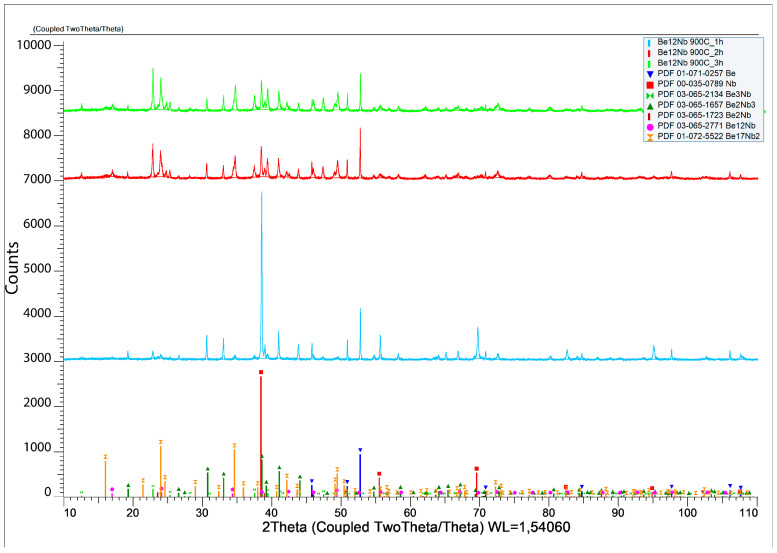
X-ray diffraction patterns after sintering at 900 °C for 1 h (blue), 2 h (red), and 3 h (green).

The results of X-ray diffraction analysis after sintering at 900 °C for 1, 2, and 3 h are presented in Figure 9.

**Figure 10:**
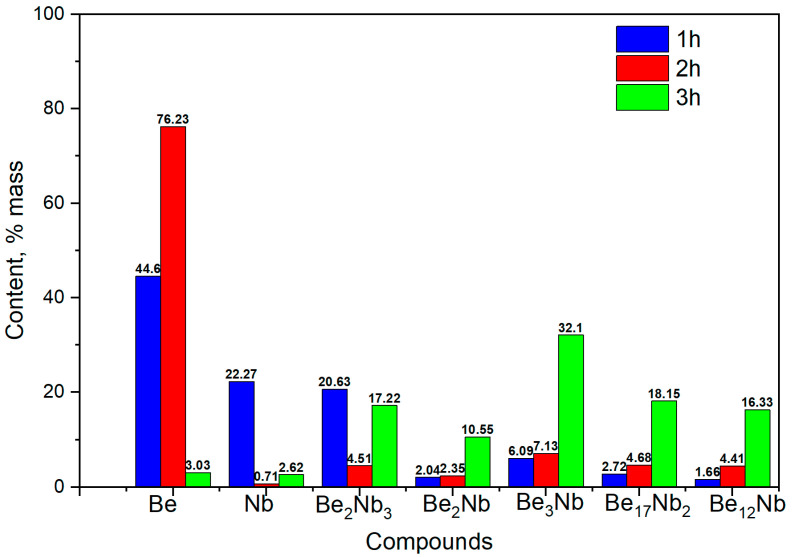
Phase composition of powders after sintering at 900 °C for 1 h (blue), 2 h (red), and 3 h (green).

The formation of several intermetallic phases is evident at this temperature. Figure 10 shows the evolution of phase composition with time. After 1 h, the contents of unreacted beryllium and niobium decrease significantly (44.6 wt.% and 22.27 wt.%, respectively), and Be_2_Nb_3_ begins to form (~20 wt.%). After 2 h, virtually no free niobium remains (~0.71 wt.%), and all five beryllide phases (Be_12_Nb, Be_17_Nb_2_, Be_3_Nb, Be_2_Nb, and Be_2_Nb_3_) are detected, each with roughly similar fractions (~4.6 wt.% average). By 3 h, the content of Be_3_Nb peaks at ~30 wt.%, with most elemental niobium and beryllium depleted. Some beryllium may remain either dissolved within the intermetallics or present as atomic-scale residue at phase boundaries.

**Figure A2:**
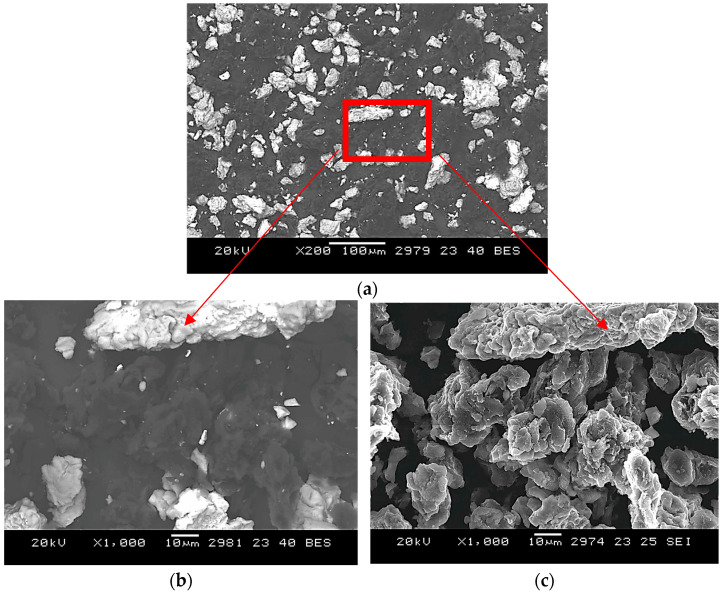
SEM images of niobium beryllide powders sintered at 900 °C for 2 h: (**a**) ×200 magnification in BSE mode; (**b**) ×1000 magnification in BSE mode; (**c**) ×1000 magnification in SE mode.

SEM analysis of powders sintered for 2 h at 900 °C (Figure A2) shows morphological changes consistent with early-stage intermetallic formation.

The niobium particles appear more rounded with smoother surfaces, suggesting the onset of diffusion-driven phase development. However, a considerable fraction of the original powders remains unreacted, indicating an incomplete transformation at this stage.

### 3.3. Synthesis at 1000 °C

**Figure 11:**
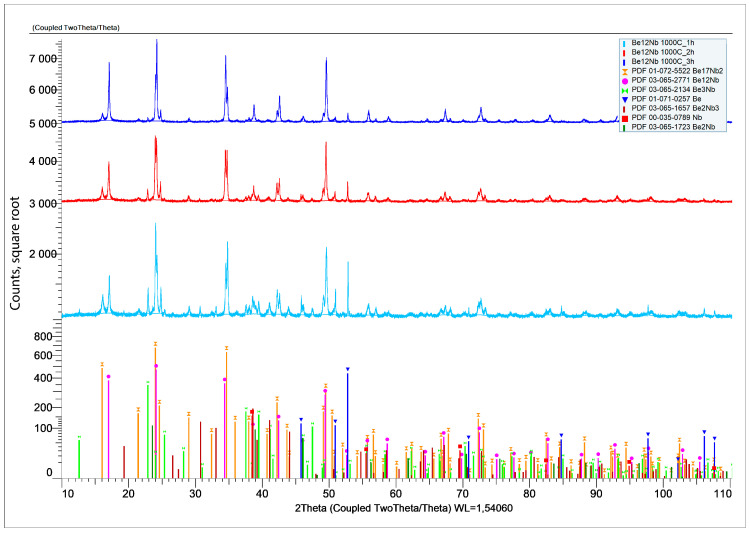
X-ray diffraction patterns after sintering at 1000 °C for 1 h (blue), 2 h (red), and 3 h (green).

The results of X-ray phase analysis after sintering at 1000 °C for 1, 2, and 3 h are shown in Figure 11.

**Figure 12:**
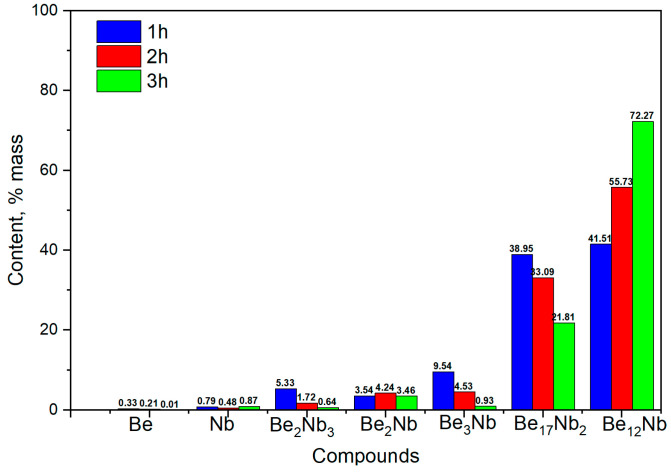
Phase composition of powders after sintering at 1000 °C for 1 h (blue), 2 h (red), and 3 h (green).

A marked shift in phase distribution is observed at this temperature. The diffraction intensity of unreacted Nb and Be significantly decreases, and a substantial rise in the intensity of Be_12_Nb and Be_17_Nb_2_ peaks is noted. Figure 12 shows the compositional dynamics of the powder mixture.

After 1 h, Be_12_Nb and Be_17_Nb_2_ each constitute approximately 40 wt.% of the mixture, while the residual elemental content drops below 1 wt.%. By 2 and 3 h, the Be_12_Nb phase continues to grow, reaching 55 wt.% and 72 wt.%, respectively, as other intermediate beryllides reduce.

### 3.4. Synthesis at 1100 °C

**Figure 13:**
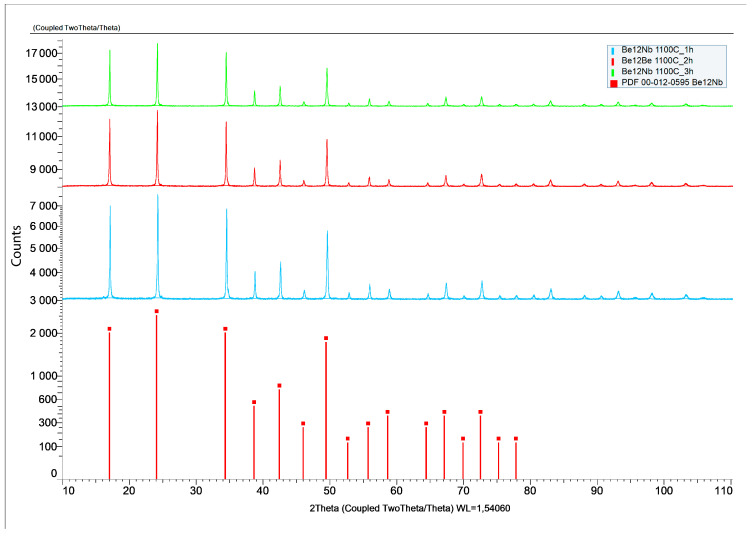
X-ray diffraction patterns after sintering at 1100 °C for 1 h (blue), 2 h (red), and 3 h (green).

The X-ray diffraction patterns of powders sintered at 1100 °C for 1, 2, and 3 h are presented in Figure 13.

**Figure 14:**
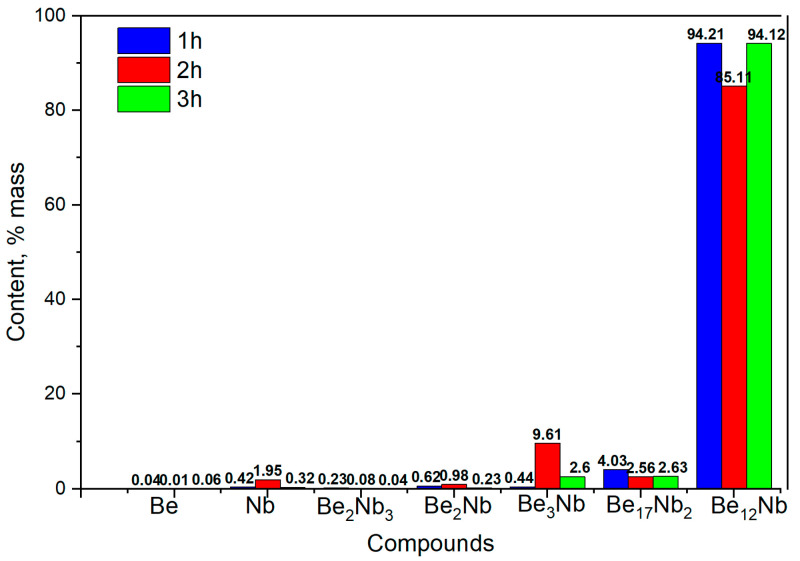
Phase composition of powders after sintering at 1100 °C for 1 h (blue), 2 h (red), and 3 h (green).

At this temperature, the phase composition becomes largely monophasic. From the first hour of sintering, Be_12_Nb is the predominant phase (~95 wt.%), with minor traces of Be_17_Nb_2_ (~4 wt.%). This trend remains consistent for extended sintering durations (2 and 3 h), indicating that 1100 °C is optimal for achieving a nearly single-phase Be_12_Nb compound. The calculated post-annealing content of the mixture components for these sintering conditions is shown in Figure 14.

**Figure A3:**
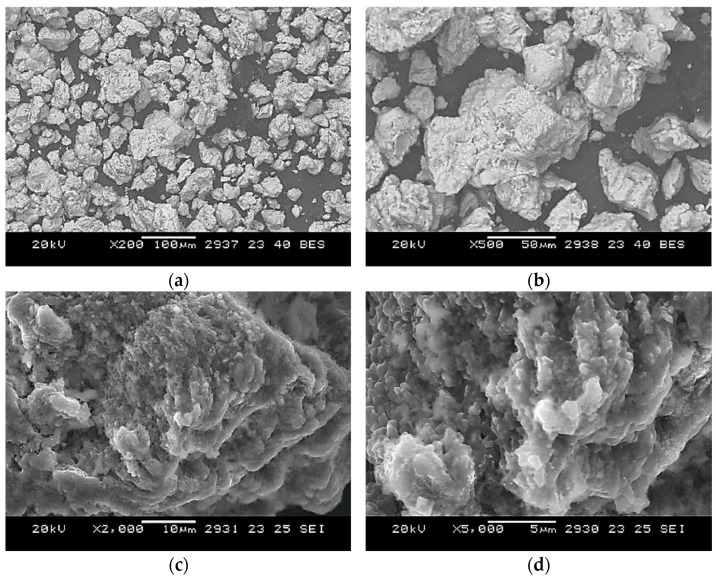
SEM images of the powder surface after sintering for 2 h at 1100 °C: (**a**) ×200 magnification in BSE mode; (**b**) ×500 magnification in SE mode; (**c**) ×2000 magnification in SE mode; (**d**) ×5000 magnification in SE mode.

SEM images of powders sintered at 1100 °C for 2 h are shown in Figure A3. The microstructure becomes more uniform compared to lower temperatures. Beryllium particles are no longer visible, and the Be_12_Nb particles appear rounded and slightly swollen, consistent with full transformation and densification.

### 3.5. Synthesis at 1200 °C

**Figure 15:**
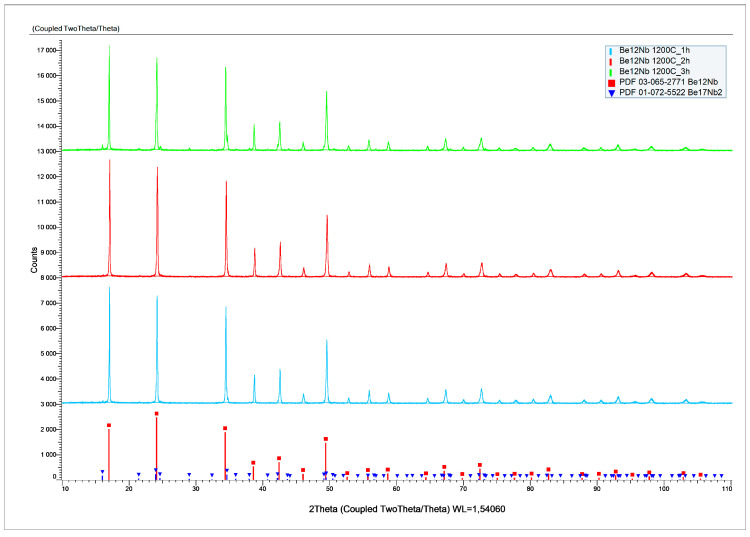
X-ray diffraction patterns after sintering at 1200 °C for 1 h (blue), 2 h (red), and 3 h (green).

The X-ray diffraction patterns of powders sintered at 1200 °C for 1, 2, and 3 h are presented in Figure 15.

**Figure 16:**
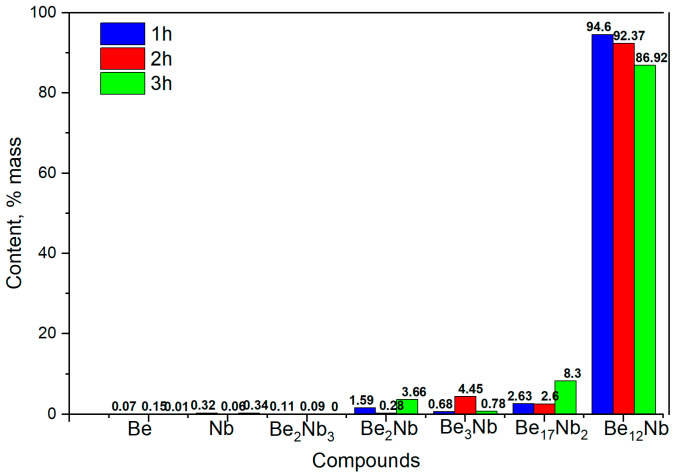
Phase composition of powders after sintering at 1200 °C for 1 h (blue), 2 h (red), and 3 h (green).

Figure 16 shows the evolution of phase composition with time. After 1 and 2 h, the mixture is predominantly monophasic with ~94 wt.% and ~92 wt.% Be_12_Nb, respectively. However, after 3 h, the Be_12_Nb content declines, and Be_17_Nb_2_ (~8 wt.%), along with other beryllides with Be/Nb < 3, begin to reappear, totaling ~5 wt.%.

The surface features sintered at 1200 °C are largely consistent with those observed at 1100 °C. While the morphology of Be_12_Nb is generally preserved, the XRD peaks are slightly weaker in intensity compared to the 1100 °C samples, possibly indicating the onset of partial decomposition or phase redistribution.

The decomposition of the niobium beryllide Be_12_Nb at temperatures of 1200 °C and above can likely be explained by the following reasons.

First, at temperatures of 1200 °C and higher under vacuum conditions, Be_12_Nb begins to dissociate due to the breaking of certain bonds within the compound, followed by the evaporation of beryllium into the free volume of the vacuum furnace.

Second, as the temperature approaches the α-Be to β-Be phase transformation, a rearrangement of the electronic structure occurs, which likely also leads to a restructuring of the crystal lattice at this temperature, resulting in the formation of weaker chemical bonds. This causes some beryllium atoms to detach and evaporate into the free volume of the furnace.

### 3.6. Synthesis at 1300 °C

**Figure 17:**
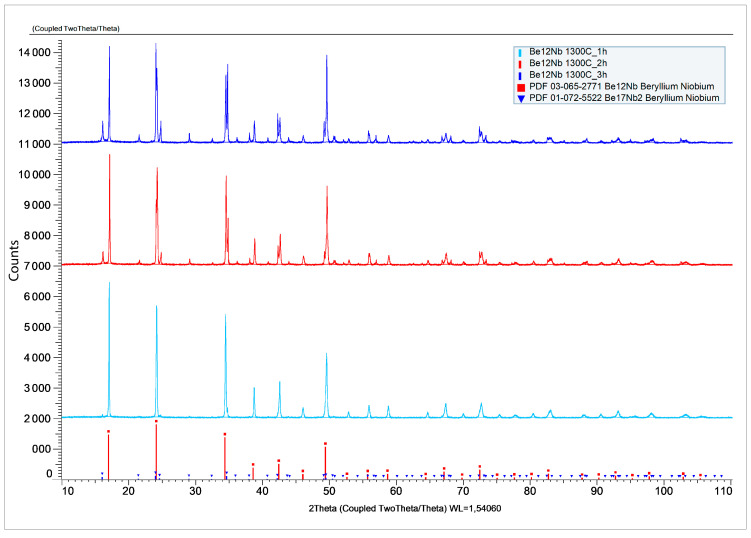
X-ray diffraction patterns after sintering at 1300 °C for 1 h (light blue), 2 h (red), and 3 h (dark blue).

The X-ray diffraction patterns after sintering at 1300 °C for 1, 2, and 3 h are shown in Figure 17.

**Figure 18:**
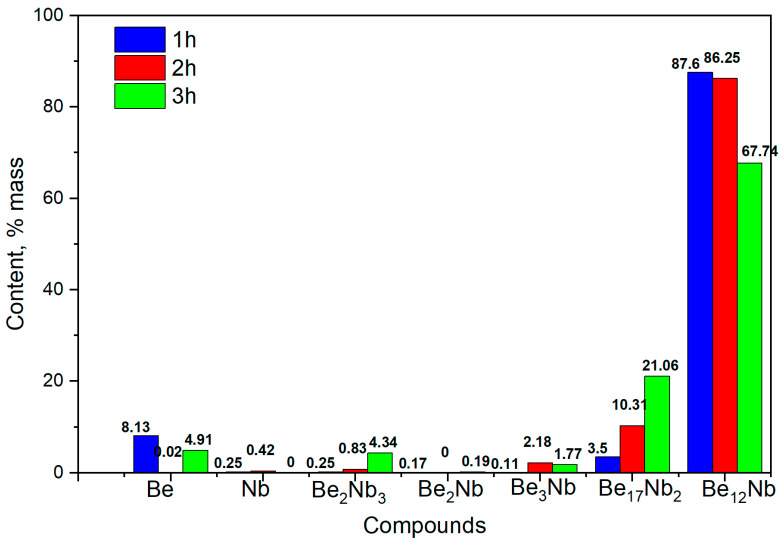
Phase composition of powders after sintering at 1300 °C for 1 h (blue), 2 h (red), and 3 h (green).

The corresponding phase composition of the synthesized powders is shown in Figure 18. At 1 h, the phase Be_12_Nb dominates (~87 wt.%), accompanied by minor amounts of Be_17_ Nb_2_ and residual beryllium (~8 wt.%). As sintering progresses to 2 h, the proportion of Be_12_Nb slightly decreases to 86 wt.%, while Be_17_Nb_2_ increases to ~10 wt.%. After 3 h, a more complex multiphase mixture emerges. Be_12_Nb content decreases substantially (~67 wt.%), and low-order beryllides and free beryllium appear more prominently, suggesting either decomposition of Be_12_Nb or incomplete transformation.

**Figure A4:**
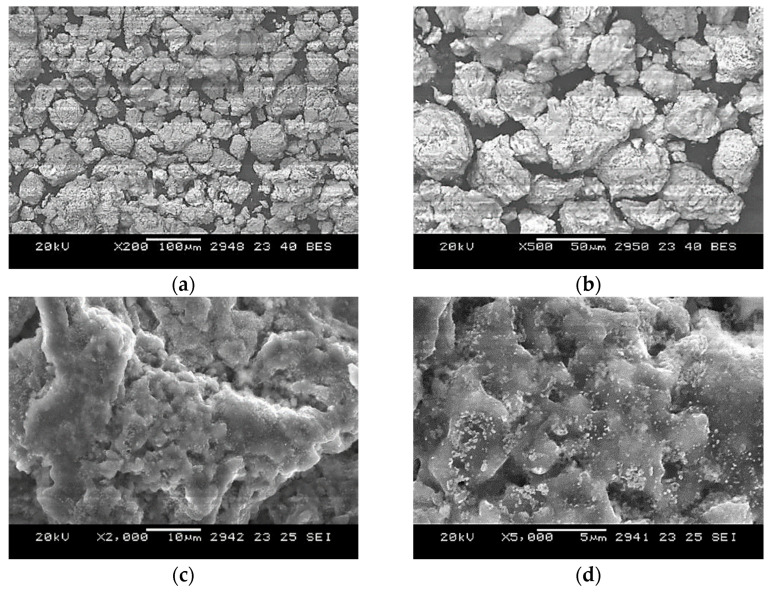
SEM images of the powder surface after sintering for 2 h at 1300 °C: (**a**) ×200 magnification in BSE mode; (**b**) ×500 magnification in BSE mode; (**c**) ×2000 magnification in SE mode; (**d**) ×5000 magnification in SE mode.

SEM analysis of powders sintered at 1300 °C for 2 h is shown in Figure A4. The microstructure is similar to that at 1200 °C, with Be_12_Nb particles still present but displaying signs of instability. Compared to the samples sintered at 1100 °C, the features are less defined, and localized degradation is more evident. This suggests a possible onset of thermal instability in the Be_12_Nb phase under vacuum conditions at this temperature.

### 3.7. Additional Synthesis at 1300 °C

Due to the increasing formation of other intermetallic phases at 1300 °C with prolonged sintering, further experiments were conducted to elucidate the underlying mechanisms. Two hypotheses were considered: (1) partial evaporation of beryllium under vacuum due to its high volatility above its melting point (1283 °C), and (2) thermal instability of Be_12_Nb itself at elevated temperatures, leading to its decomposition into lower-order beryllides.

**Figure 19:**
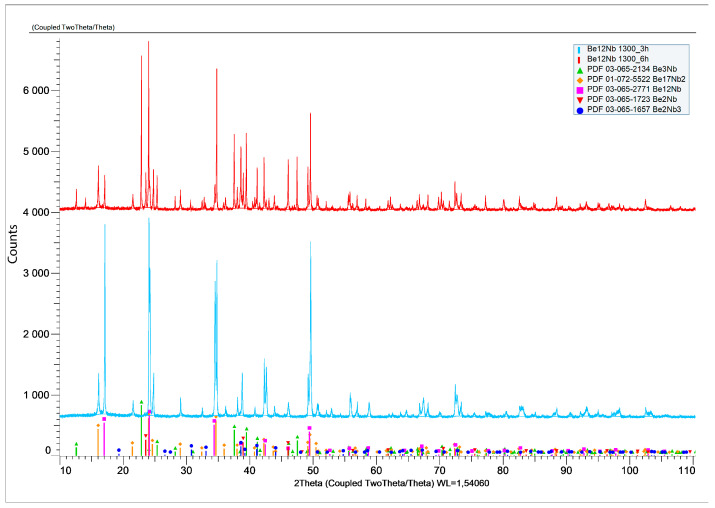
X-ray diffraction patterns after sintering at 1300 °C for 3 h (light blue) and 6 h (dark blue).

To test these possibilities, fresh powder mixtures were compacted and sintered at 1300 °C for 3 and 6 h. XRD results (Figure 19) showed a significant decrease in Be_12_Nb content, particularly after 6 h. For 3 h of sintering, Be_12_Nb was still the dominant phase (83 wt.%), but after 6 h, Be_17_Nb_2_ became predominant (57 wt.%), while Be_12_Nb dropped to 17 wt.%.

**Figure A5:**
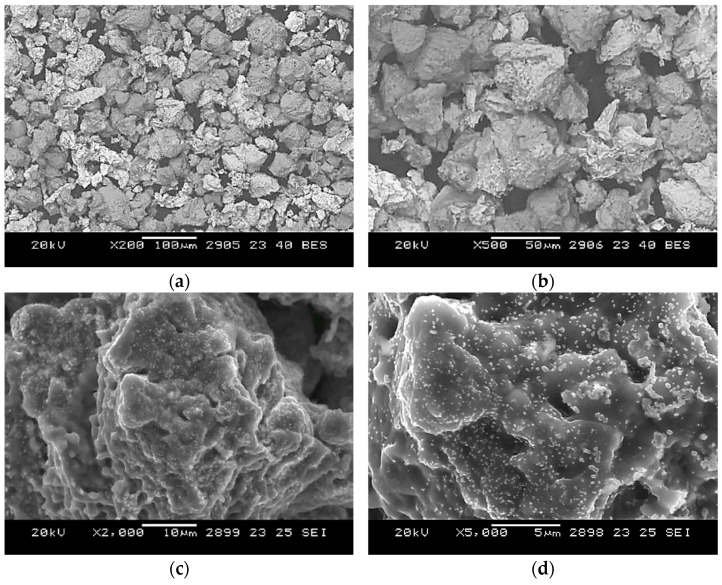
SEM images of the powder surface after sintering for 6 h at 1300 °C: (**a**) ×200 magnification in BSE mode; (**b**) ×500 magnification in BSE mode; (**c**) ×2000 magnification in SE mode; (**d**) ×5000 magnification in SE mode.

SEM images of the powder sintered for 6 h at 1300 °C are presented in Figure A5, revealing structural degradation consistent with phase transformation and beryllium loss.

**Figure 20:**
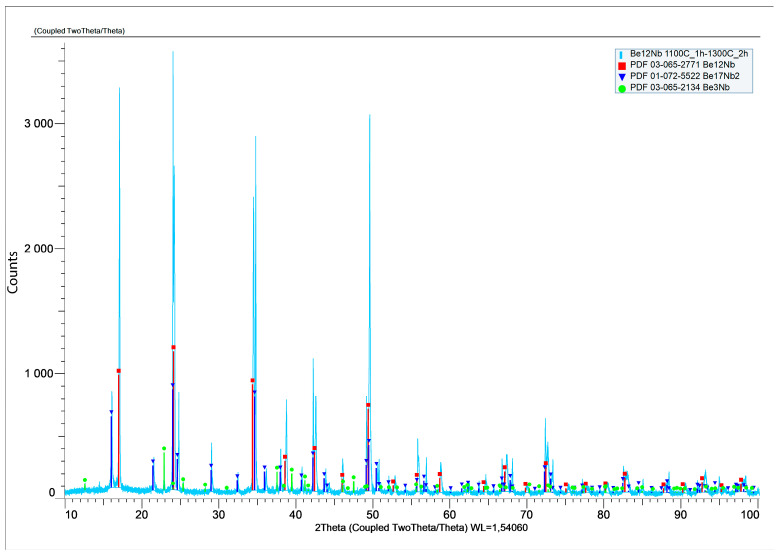
XRD patterns after stepwise sintering at 1100 °C for 1 h, followed by 1300 °C for 2 h.

A two-step sintering process was also performed to separate thermal effects from beryllium volatilization. The sample was held at 1100 °C for 1 h and subsequently sintered at 1300 °C for 2 h. XRD analysis (Figure 20) expressed in logarithmic units confirmed a heterogeneous phase composition, with 59 wt.% Be_12_Nb, 34 wt.% Be_17_Nb_2_, and minor fractions of Be_3_Nb and Be_2_Nb.

**Figure 21:**
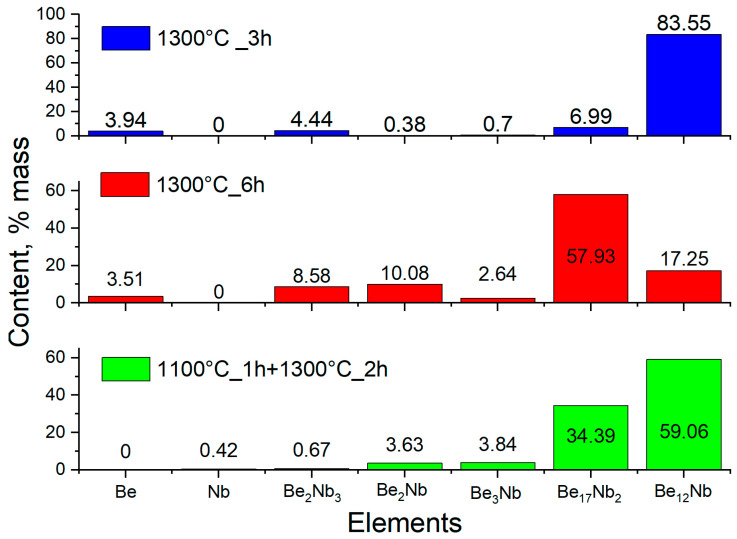
Phase composition of niobium beryllide powders after stepwise sintering.

The phase distribution for this two-step process is illustrated in Figure 21.

These findings suggest that Be_12_Nb becomes thermally unstable at or above 1300 °C under vacuum, leading to its dissociation into lower-order beryllides.

## 4. Discussion

**Figure 22:**
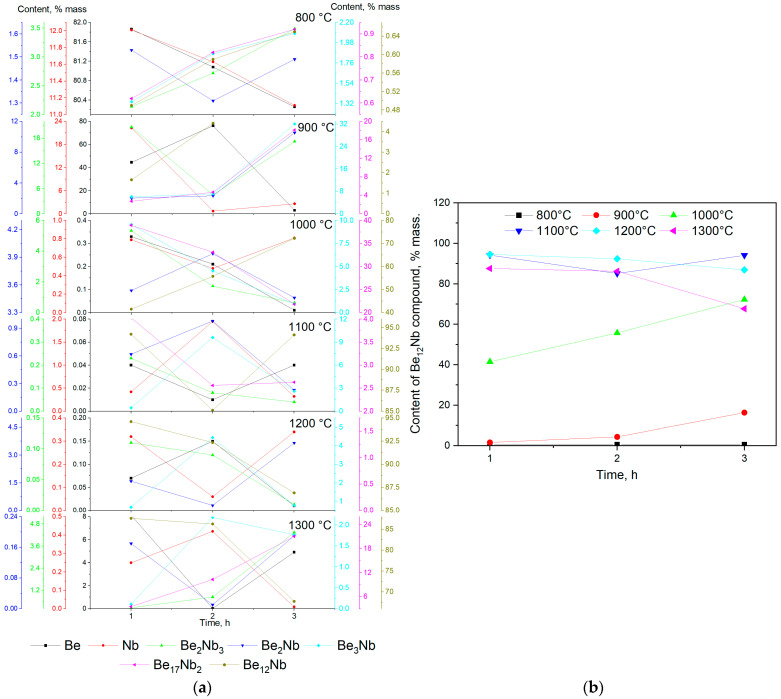
Summarized outcomes of the Be_12_Nb synthesis: (**a**) Temperature–time dependence during the synthesis of niobium beryllide Be_12_Nb from beryllium and niobium powders; (**b**) dynamic evolution of Be–Nb intermetallic phases synthesized in the 800–1300 °C range.

Figure 22 summarizes the outcomes of the synthesis experiments, illustrating the evolution of various intermetallic phases, including the target compound Be_12_Nb, as a function of sintering temperature and time. At the initial stage (800 °C), the XRD patterns and quantitative analysis show that the samples primarily consist of unreacted niobium and beryllium powders. The synthesis of intermetallic phases is minimal and limited to low levels of Be_2_Nb_3_, indicating that the activation energy required for significant diffusion and reaction is not achieved at this temperature.

By 900 °C, the onset of intermetallic compound formation becomes evident, starting with the Be_2_Nb_3_ phase via the reaction pathway 3Nb + 2Be → Be_2_Nb_3_. This reaction occurs well below the melting points of either constituent, suggesting a solid-state diffusion mechanism. The small atomic radius and higher mobility of beryllium facilitate its penetration into the niobium lattice, leading to progressive intermetallic formation from the particle surfaces inward. The formation front likely begins at the beryllium–niobium interface and progresses through successive reactions, forming intermediate compounds such as Be_2_Nb and Be_3_Nb. These phases represent different local Be/Nb ratios depending on beryllium availability at the reaction interface. The formation of these intermediate phases continues as the system evolves towards equilibrium.

At 1000 °C, the Be_12_Nb phase becomes more prominent, but full conversion is not achieved within the studied time frames. Residual Be_17_Nb_2_ and trace beryllium suggest incomplete transformation or possible precursor–product relationships between Be_12_Nb and other phases. It has been proposed in earlier work [35] that Be_17_Nb_2_ may result from the decomposition or transformation of Be_12_Nb under certain thermal conditions, possibly explaining their co-existence.

The most favorable condition for the formation of a nearly single-phase Be_12_Nb material was achieved at 1100 °C. At this temperature, Be_12_Nb accounts for more than 95 wt.% of the sample, indicating optimal kinetics for diffusion and reaction. The formation mechanism likely involves sequential incorporation of beryllium into niobium-rich phases until the stoichiometric 12:1 ratio is satisfied.

At 1200 °C, however, the system begins to show early signs of instability. Although Be_12_Nb is still the predominant phase after 1–2 h, longer exposure leads to the emergence of Be_17_Nb_2_ and other lower-order beryllides. This suggests that elevated temperatures may induce phase dissociation, particularly under low vacuum conditions where beryllium volatility increases.

At 1300 °C, the thermal stability of Be_12_Nb further degrades. As demonstrated by both standard and extended sintering trials, prolonged exposure results in a significant decline of Be_12_Nb content, replaced by Be_17_Nb_2_ and other low-order phases. This behavior may result from the evaporation of beryllium and/or the thermal decomposition of Be_12_Nb.

The stepwise sintering experiment (1100 °C for 1 h followed by 1300 °C for 2 h) reinforces this hypothesis. Despite the initial formation of Be_12_Nb at 1100 °C, subsequent exposure to 1300 °C resulted in its partial degradation and the formation of Be_17_Nb_2_, Be_3_Nb, and Be_2_Nb.

Comparison with prior studies confirms these findings. Nakamichi et al. [21] and Kim J.-H. et al. [22] reported that while sintering at 900–1000 °C enables Be_12_Nb formation, extended times are required, and complete phase purity is not achieved. Our results demonstrate that sintering at 1100 °C is more effective, allowing for higher phase purity in shorter durations. Furthermore, our study reveals the critical limitation of Be_12_Nb at elevated temperatures, a detail not emphasized in prior reports.

In summary, the findings from this study clarify the phase formation pathway of Be_12_Nb and identify 1100 °C as the optimal sintering temperature for achieving a nearly single-phase material. Beyond this temperature, thermal instability under vacuum leads to degradation, limiting the processing window for potential applications.

## 5. Conclusions

This study provides a comprehensive analysis of the phase evolution during the synthesis of the Be_12_Nb intermetallic compound from a mixture of pre-compacted beryllium and niobium powders across the temperature range of 800–1300 °C. The experimental findings confirm that:

- Intermetallic phase formation initiates at 900 °C, beginning with Be_2_Nb_3_, and proceeds through intermediate phases as beryllium diffuses into the niobium matrix.
- The optimal synthesis temperature for nearly single-phase Be_12_Nb formation is 1100 °C, where over 95 wt.% Be_12_Nb was achieved after 1–3 h of sintering.
- Temperatures above 1200 °C lead to the decomposition of Be_12_Nb and the formation of lower-order beryllides such as Be_17_Nb_2_, likely due to thermal instability and beryllium volatility under vacuum.
- Two-stage and prolonged sintering experiments confirm the metastable nature of Be_12_Nb at elevated temperatures, emphasizing the importance of thermal control during processing.

This information will help substantiate the optimal processing parameters for producing Be_12_Nb-based billets and guide the technological considerations necessary for fabricating components with stable phase composition via optimized sintering conditions.

## Figures and Tables

**Table 1:** Chemical composition of PTB-56 grade beryllium powder.

Element	Be	Cr	Mg	Si	Ti	Fe	Ni	Pb	Al	Cu	C	O	Mn
Content (%, wt.)	99.107	0.023	0.014	0.024	0.014	0.156	0.017	0.01	0.018	0.003	0.04	0.56	0.014

**Table 2:** Chemical composition of NbP-3b grade niobium powder.

Element	Nb	Ta	Ti	Si	Fe	W	Mo	N	C	O	H	Ni	Al	Mg	Cu	Zr
Content (%, wt.)	99.706	0.09	0.001	0.002	0.002	0.003	0.004	0.012	0.007	0.13	0.008	0.001	0.001	0.001	0.001	0.001

## Data Availability

The original contributions presented in this study are included in the article. Further inquiries can be directed to the corresponding author.

## Abbreviations

The following abbreviations are used in this manuscript:

ITER	International Thermonuclear Experimental Reactor
DEMO	Demonstration power plant
XRD	X-ray powder diffraction
SEM	Scanning electron microscope
SE	Secondary electrons
BSE	Backscattered electrons

## Appendix A

**Table A1:** Crystal structures of Be-Nb intermetallic compounds at 20 °C.

Compound	Proportion of Be in the Compound	Pearson Symbol, Space Group	Crystal Lattice Parameters	ICDD PDF2 Card Numbers
Be	100% wt.	Hexagonal, hP2, P63/mmc	a = b = 2.2858, c = 3.5843 α = β = 90°, γ = 120° V_cr. lat._ = 16.22	01-071-0257
Nb	0% wt.	Cubic, cI2, Im-3m	a = b = c = 3.3066, α = β = γ = 90° V_cr. lat._ = 36.15	00-035-0789
Be_2_Nb_3_	6.07% wt.	Tetragonal, tP10, P4/mbm	a = b = 6.490, c = 3.350, α = β = γ = 90° V_cr. lat._ = 141.10	03-065-1657
Be_2_Nb	16.25% wt.	Cubic, cF24, Fd3m	a = b = c = 6.535, α = β = γ = 90° V _cr. lat._ = 279.09	03-065-1723
Be_3_Nb	22.54% wt.	Rhombohedral, hR12, R-3m	a = b = 4.561, c = 21.050 α = β = γ = 90° V _cr. lat._ = 379.23	03-065-2134
Be_17_Nb_2_	45.19% wt.	Rhombohedral, hR19, R3m	a = b = 7.409, c = 10.840 α = β = 90°, γ = 120° V _cr. lat._ = 515.32	01-072-5522
Be_12_Nb	53.79% wt.	Tetragonal, tI26, I4/mmm	a = b = 7.3272, c = 3.350 α = β= γ = 90° V _cr. lat._ = 231.30	03-065-2771
